# Incidental Identification of Vertebral Fragility Fractures by Chest CT in COVID-19-Infected Individuals

**DOI:** 10.7759/cureus.24867

**Published:** 2022-05-09

**Authors:** Vishal Patil, Ayapaneni Dileep Reddy, Amit Kale, Abhinay Vadlamudi, Janapamala V S Kishore, Chiranjivi Jani

**Affiliations:** 1 Orthopaedics, Dr. D. Y. Patil Medical College, Hospital & Research Centre, Pune, IND; 2 Radiology, Dr. D. Y. Patil Medical College, Hospital & Research Centre, Pune, IND

**Keywords:** chest ct, covid-19 india, fragility fractures, osteoporosis, vertebral compression fractures

## Abstract

Introduction

It is critical to identify asymptomatic vertebral compression fractures (VCFs) as soon as possible in order to avoid subsequent fragility fractures. The purpose of the study was to see how many vertebral compression fractures there were in patients admitted to the COVID-19 pneumonia unit in a single tertiary care hospital who underwent chest computed tomography (CT) scans.

Materials and methods

Sagittal reconstruction of the thoracic spine was done in around 504 patients and classified into mild, moderate, and severe categories, and we compared it with the radiological reports of the same.

Results

In our study, the median age was 53 years (range: 31-91 years); 63% were men and 37% were women. Of the 504 patients, 76 (15%) had at least one vertebral compression fracture (VCF); 53 (10.2%) had one VCF, and 23 (4.8%) had multiple VCF, with 50 having mild fractures, 15 having moderate fractures, and 11 having severe fractures. Males (13.87%) and females (14.72%) had the same proportion of VCF (p = 0.83). Only 10% of the patients with VCFs we identified had a description in their report (eight patients).

Conclusion

The reporting of VCF is insufficient. VCF detection should be included in the search patterns of radiologists and physicians, regardless of the primary reason for performing chest CT. Although many patients are unable to come to the hospital during pandemic/epidemic, careful evaluation and inclusion of mild fractures in reports, as well as an explanation of the risk of subsequent fractures and treatment accordingly, would completely eliminate the risk of subsequent fractures.

## Introduction

Osteoporosis is a global problem that is most usually discovered by vertebral fractures, and the presence of vertebral compression fractures (VCFs) are indicators of osteoporosis in an otherwise unremarkable history and may produce non-vertebral osteoporotic fractures without additional intervention [1-6]. Almost a quarter of the senior population has reported spinal compression fractures in chest radiographs from earlier research, and numerous studies frequently cite underreporting of these fractures [7-11]. The inadvertent diagnosis of vertebral compression fractures in non-back pain-presenting individuals is critical for reducing the risk of fragility fractures [12].

Although computed tomography (CT) is an excellent diagnostic technique for both finding and measuring fractures, earlier research relied on midline sagittal reformatted images to diagnose fractures [13,14]. The purpose of this study was to examine if there were any vertebral compression fractures in the whole thoracic spine in patients who had received multi-detector chest CT scans for COVID-19 pneumonia severity rating, and the radiological reports were reviewed.

## Materials and methods

This retrospective investigation was authorized by the Institutional Ethics Sub-Committee (IESC) of Dr. D. Y. Patil Medical College, Hospital & Research Centre in Pune (research permission number: IESC/FP/2021/41). We conducted this investigation on 504 patients hospitalized in the COVID-19 pneumonia unit, all of whom tested positive for SARS-CoV-2 RT-PCR. This study was conducted from January to July 2021. All patients with chest CT conducted within the aforementioned time period were included, regardless of age group, and the initial scan was evaluated in patients with numerous scans. Fresh fractures and pathological fractures, which can be detected by loss of trabeculation and step defect, thoracic spine instrumentation, and CT images without full visibility of the thoracic spine in sagittal reformats, were all eliminated.

Sagittal images were created by reformatting axial images with bone windows and a section thickness of 1 mm. If needed, coronal pictures were also accessible. The CT images were examined using the Siemens software tool (PACS), which is accessible at our institution; assessors were blinded to the patients' clinical and personal data, and bone contrast windows were used. The approach of Genant et al. was used for evaluation, and patients with vertebral compression fractures were assessed by the senior author and categorized as mild (20%-25%), intermediate (25%-40%), or severe (>40%) based on vertebral height decrease [15].

**Figure 1:**
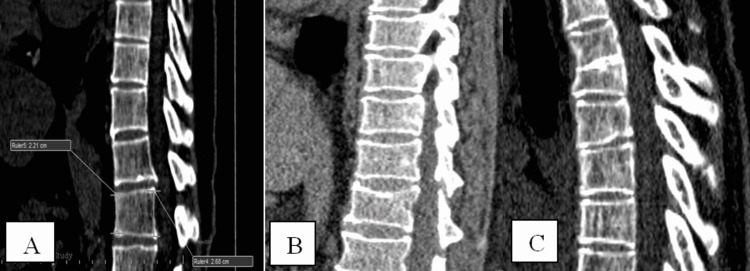
Mild anterior (A), middle (B), and posterior (C) wedging of the vertebra

Figure 1A displays the anterior wedging of the vertebrae determined using the Siemens software tool for estimating the decrease of vertebral body height from a sagittal reformatting of the bone window of 1 mm thickness. Figure 1B is a sagittal reformatted picture of a single participant from the population research with minor middle wedging of numerous vertebrae. In a sagittal reformatted picture, Figure 1C shows the modest posterior wedging of the vertebrae.

**Figure 2:**
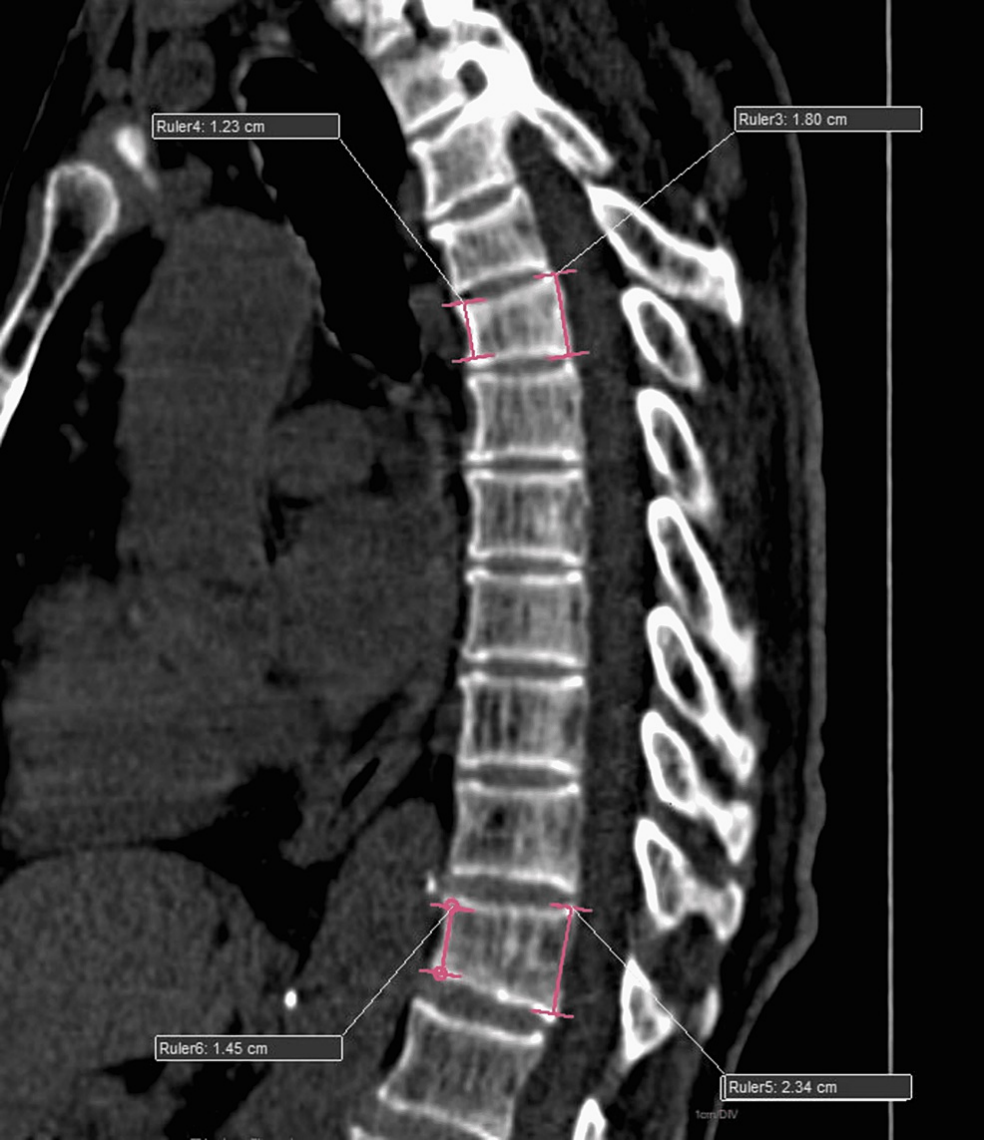
Moderate anterior wedging of the vertebra

Figure 2 depicts moderate anterior vertebral wedging in a sagittal reformatted picture.

**Figure 3:**
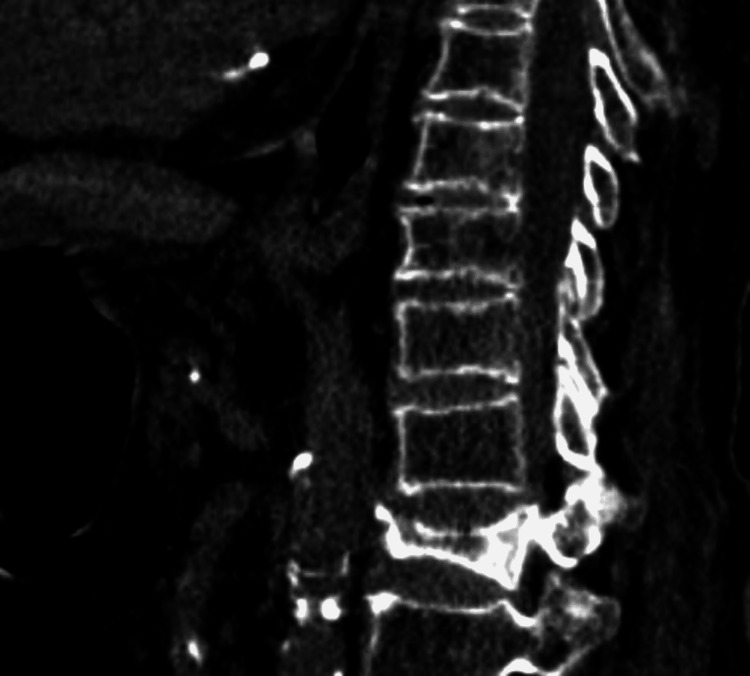
Severe anterior, middle, and posterior wedging of the vertebra

Figure 3 depicts the severe wedging that can be observed in all sections of a sagittal reformatted picture.

## Results

We examined 504 individuals, the average age being 54.3 years (range: 31-91 years). In all, 317 (63%) patients were male, while 187 (37%) were female. When comparing age group variability, the age group less than 50 accounts for 42% of the population, and the age group greater than 60 accounts for 38% of the research. There were 76 (15%) individuals who had at least one vertebral fracture. In particular, 53 (10.2%) patients had a single fracture, whereas 23 (4.8%) patients had two or more VCF, for a total of 106 fractures. T11 and T12 vertebrae accounted for more than two-thirds of the accidental vertebral compression fractures.

Table 1 shows the fracture severity in the study population, with mild fractures accounting for two-thirds of the total fractures and moderate and severe fractures accounting for the remaining third.

**Table 1:** Severity of fracture

Severity	Number	Percentage
Mild	50	65.8
Moderate	15	19.7
Severe	11	14.5
Total	76	100

Table 2 shows the levels of fracture pattern and their relationship with age group, as well as the respective p-value following comparison.

**Table 2:** Type of fracture and gender Chi-square p-value = 0.9 (not significant)

Fracture	Female		Male	
	n	%	n	%
Single level	21	39.6	32	60.4
Multiple levels	9	39.1	14	60.8
Total	30	100	46	100

Table 3 shows the age distribution of persons who have fractures.

**Table 3:** Distribution of age group among those having fractures

Age categories	Number	Percentage
Less than 50	18	23.7
50-60	16	21.1
More than 60	42	55.3
Total	76	100

The column distribution among the fractures is shown in Table 4.

**Table 4:** Distribution of columns among those who had a fracture

Column	Number	Percentage
Anterior	39	51.3
Middle	13	17.1
Posterior	1	1.3
Anterior and middle	20	26.3
Anterior, middle, and posterior	3	4
Total	76	100

**Figure 4:**
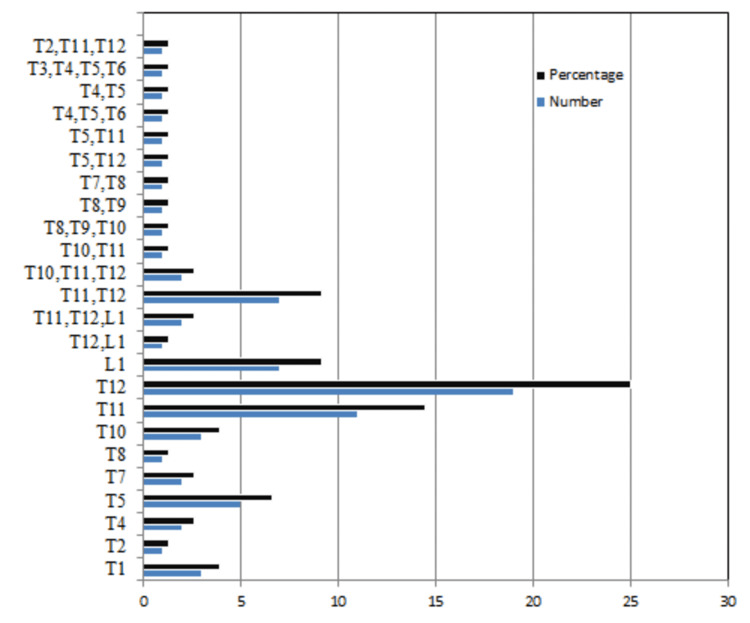
Distribution of level of fracture among those who had a fracture

Figure 4 depicts the level of thoracic vertebrae in spinal compression fractures, with the thoracolumbar junction accounting for nearly two-thirds of the total fractures.

## Discussion

The International Osteoporosis Foundation has concluded that one best practice standard for health institutions is the development of a VCF incidental detection methodology [16]. Many physicians relate the reasons for back pain to other disorders, which might lead to the underdiagnosis of a serious systemic condition, osteoporosis [17].

Our study is the first in the COVID-19 era to evaluate osteoporotic vertebral compression fractures using sagittal reformats of chest CT. Many previous studies compared the relationship between osteoporosis and COVID-19 infectivity, but none of them evaluated osteoporotic vertebral compression fractures in sagittal reformats of the vertebrae in chest CT [18]. Our study found a 15% incidence of vertebral compression fractures, which is consistent with earlier studies that found a 12%-18% incidence of vertebral compression fractures in asymptomatic adults with no substantial prior history or comorbidities [19]. The aforementioned figure is based mostly on the population over the age of 50, and if the younger age groups are excluded, the incidence rises to more than 25%, as evidenced by earlier studies that assessed fractures using sagittal reformats [20].

Also, the proportion of compression fractures observed in the male population is higher than in the female population, and the number of multiple fractures observed in women is higher than in men, despite the fact that the value did not achieve a significant p-value. This can be ascribed to the study's huge male-dominant population, which made up over two-thirds of the population, in contrast to earlier research that found osteoporotic vertebral compression fractures to be more prevalent in female age groups [21]. The age of vertebral fragility fractures rises with age, and around 60% of the fractures are reported above the age of 60, which matches prior research revealing comparable statistics of VCFs [22].

Only a 10th of the observed fractures are acknowledged in reports, with radiologists reporting only eight of the 76 fractures, all of which are severe compression fractures seen on sagittal slices of chest CT before reformatting the bone images. The clinical scenario in these cases, where radiologists are keen on giving CORADS score and also looking out for potential other causes, such as embolism and pleural effusion, can contribute to the low reporting, and many other studies showed similar underreporting, noted in patients being evaluated for cancer and terminal illness in the ICU. Many people are avoiding hospitals in these epidemic times for general diseases such as back pain, and many individuals are limiting themselves to home without sufficient physical activity and exposure to daylight, making them more prone to osteoporosis.

Limitations

Prior history of trauma and the date of fracture were not determined because the present study is a retrospective study. Using a sagittal and coronal reformatting, vertebral deformities such as Scheuermann's disease cannot be ruled out, and CT concentrated on the dorsal spine and the lumbar spine is missing, which is an important factor because osteoporotic vertebral compression fractures are common at the dorso-lumbar junction.

Although the study was conducted on a COVID-19-afflicted population, this cannot be considered the only constraint because the scan was completed until the L1 vertebrae, which covers almost the whole thoracolumbar junction. Age group can also be a limiting factor since vertebral compression fractures are common in the older age group and here all patients are taken into study irrespective of the age group.

## Conclusions

Underreporting of vertebral compression fractures is very evident in the above study, and radiologists should look for vertebral compression fractures regardless of their primary reason. During these pandemic/epidemic times, osteoporosis is a neglected disease, leading to underdiagnosis and undertreatment. As a result, a thorough examination of spinal compression fractures, including even mild to moderate fractures in reports, and emphasizing the danger of additional fractures are required. Treating suitably during the same hospital visit, not only in COVID-19-infected patients receiving chest CT such as in our study but also in other tests such as abdominal CT conducted during cancer staging and many others, would eliminate the danger entirely and avoid morbidity in many patients.
